# Classification of female MDD patients with and without suicidal ideation using resting-state functional magnetic resonance imaging and machine learning

**DOI:** 10.3389/fnhum.2024.1427532

**Published:** 2025-01-08

**Authors:** Morteza Fattahi, Milad Esmaeil-Zadeh, Hamid Soltanian-Zadeh, Reza Rostami, Jamil Mansouri, Gholam-Ali Hossein-Zadeh

**Affiliations:** ^1^School of Electrical and Computer Engineering, College of Engineering, University of Tehran, Tehran, Iran; ^2^School of Cognitive Science, Institute for Research in Fundamental Sciences (IPM), Tehran, Iran; ^3^Departments of Radiology and Research Administration, Henry Ford Health System, Detroit, MI, United States; ^4^School of Psychology and Education, University of Tehran, Tehran, Iran; ^5^School of Psychology and Education, Kharazmi University, Karaj, Iran

**Keywords:** resting-state fMRI, major depressive disorder, suicide ideation, feature selection, Random Forest Classifier, elastic net, default mode network, Central Executive Control Network

## Abstract

Spontaneous blood oxygen level-dependent signals can be indirectly recorded in different brain regions with functional magnetic resonance imaging. In this study resting-state functional magnetic resonance imaging was used to measure the differences in connectivity and activation seen in major depressive disorder (MDD) patients with and without suicidal ideation and the control group. For our investigation, a brain atlas containing 116 regions of interest was used. We also used four voxel-based connectivity models, including degree centrality, the fractional amplitude of low-frequency fluctuations (fALFF), regional homogeneity, and voxel-mirrored Homotopic Connectivity. Feature selection was conducted using a sequential backward floating selection approach along with a Random Forest Classifier and Elastic Net. While all four models yield significant results, fALFF demonstrated higher accuracy rates in classifying the three groups. Further analysis revealed three features that demonstrated statistically significant differences between these three, resulting in a 90.00% accuracy rate. Prominent features identified from our analysis, with suicide ideation as the key variable, included the Superior frontal gyrus (dorsolateral and orbital parts), the median cingulate, and the paracingulate gyri. These areas are associated with the Central Executive Control Network (ECN), the Default Mode Network, and the ECN, respectively. Comparing the results of MDD patients with suicidal ideation to those without suicidal ideations suggests dysfunctions in decision-making ability, in MDD females suffering from suicidal tendencies. This may be related to a lack of inhibition or emotion regulation capability, which contributes to suicidal ideations.

## 1 Introduction

The presence of pain and hopelessness can contribute to the development of suicidal ideation. Individuals experiencing these feelings may be more prone to considering suicide as a possible solution. It is important to take these emotional challenges seriously and provide appropriate support to those in need (Klonsky et al., 2016). Major Depressive Disorder (MDD) is one of the most common mental illnesses frequently linked to suicidal ideation and behavior (Heuschen et al., 2022; Ribeiro et al., 2018; Baldessarini and Tondo, 2020). Misdiagnosing a disorder and administering drugs based on that misdiagnosis can amplify the risk of suicidal ideation (SI) among patients with mood disorders such as MDD. For instance, the use of serotonin-selective reuptake inhibitors, commonly prescribed for unipolar depression, can heighten SI in patients with bipolar disorder (Hogg et al., 2016), making it crucial to identify the specific type of disorder and administer appropriate treatment.

Functional Magnetic Resonance Imaging (fMRI) has become a popular method in recent years for studying the brain, which is an efficient network of many areas that have their intrinsic function while also communicating and transferring information with each other (Van Den Heuvel and Pol, 2010). Numerous studies have been conducted to investigate brain segregation and integration (Friston, 2011). The brain balances the segregation and integration of incoming stimuli to regulate the flow of information (Deco et al., 2015). To measure this information, two types of fMRI data can be used: task-based fMRI and resting-state fMRI (Chen and Glover, 2015). Resting-state fMRI measures spontaneous low-frequency fluctuations in the Blood Oxygen Level Dependent (BOLD) signal, which can be used to explore the functional organization of the brain. Resting-state fMRI has become a more common method since (1) the same data can be used to study the human brain in various projects; (2) resting-state fMRI consumes more energy than in task-based fMRI, meaning that rest fMRI captures abundant neuronal signals (Daliri and Behroozi, 2013).

Functional connectivity provides insight into the brain signals and data communication within the brain networks (Shahhosseini and Miranda, 2022). Certain regions of the brain exhibit flexible interactions to support a wide range of cognitive functions. These interconnected regions are collectively referred to as brain networks (Pessoa, 2014). Analyzing brain networks reveals vital topological properties. In MDD with suicide ideation, the Frontoparietal network and Default Mode Network (DMN) have shown distinct connectivity patterns (Xu et al., 2022; Dai et al., 2022). The frontoparietal network is a control network, enabling rapid task state instantiation through flexible interactions with control and processing networks (Liu et al., 2021; Dai et al., 2022). DMN is a brain network that tends to activate when individuals are not focused on the external environment (Li et al., 2014).

The salience network (SN) refers to a group of brain regions that play a crucial role in determining which stimuli warrant our attention. This network is significant in the study of mental disorders like MDD (Schimmelpfennig et al., 2023). To investigate these conditions, resting-state fMRI has provided valuable insights across various clinical applications (Fox and Greicius, 2010), such as Alzheimer's disease, epilepsy, and MDD (Lee et al., 2013). Considering SN, DMN, and frontoparietal networks as target areas, seed-based analysis which uses connectivity metrics to assess the connectivity patterns within a pre-defined seed or Region of Interest (ROI), and Independent Component Analyses (ICA). ICA identifies a set of statistically independent spatial maps and their corresponding time courses (Rosazza et al., 2012; Pessoa, 2014). These methodologies have been employed to identify differences in connectivity patterns of resting-state fMRI data in bipolar and MDD (Rai et al., 2021). Graph analysis is another approach in which brain regions are considered as nodes and connections between these regions are represented as edges. It has been used as an alternative to both ICA and seed-based analysis (Rajamanickam, 2020). This approach has been applied to the rs-fMRI data from medication-free MDD patients to identify individuals exhibiting suicidal behavior (Chen et al., 2021). Another study applied ICA and seed-based correlation methods to analyze the DMN, Executive Control Networks (ECN), and SN, revealing meaningful differences in connectivity within and between these networks among MDD patients compared to normal controls (Mulders et al., 2015). Data-driven methods such as Fractional Amplitude of Low-Frequency Fluctuations (fALFF) (Gao et al., 2021), Regional Homogeneity (ReHo) (Jiang and Zuo, 2016), Degree Centrality (DC) (Shi et al., 2023), and Voxel-Mirrored Homotopic Connectivity (VMHC) (Fan et al., 2018) are famous d methods in this field. A recent study applying these methods to BOLD fMRI of the brain in patients with MDD and their first-degree relatives demonstrated distinct values of ReHo, ALFF, and fractional ALFF (fALFF) in MDD, first-degree relatives, and healthy controls (Song et al., 2020). These methodologies highlight differences between the groups, as a higher level of ReHo indicates greater regional synchronization, while fALFF is particularly effective in revealing areas such as DMN and the frontal lobe within groups. The abnormal activation patterns observed in MDD and their first-degree relatives support the hypothesis that these abnormal activities are associated with pathological mechanisms and emotional distress in MDD (Song et al., 2020).

As mentioned in previous studies, voxel-based connectivity procedures can provide higher spatial resolution than seed-based methods, which are typically limited to a single seed region. These voxel-based methods can provide more robust estimates of connectivity by averaging across multiple voxels, rather than relying on a single seed region. Furthermore, these methods can identify subtle connectivity patterns that seed-based approaches may overlook, often due to their bias toward stronger connections. Also, the ICA method has some drawbacks, including high computational complexity, the inherent problem of component permutation, the need for manual identification of independent components related to artifacts, and longer processing times. Voxel-based techniques are particularly effective for investigating large-scale brain networks, as they can capture complex connectivity patterns across multiple brain regions (Chauhan and Choi, 2022; Behrens et al., 2003; Ashburner and Friston, 2000; Vandenberghe et al., 2018; Liu et al., 2010; Naik and Kumar, 2007; Djuwari et al., 2006).

As described earlier, ReHo, DC, fALFF, and VMHC are voxel-based connectivity methods that provide advantages over seed-based and data-driven techniques such as ICA, as they are not prone to the biases mentioned previously. In this study, we employ these feature extraction methods along with machine learning tools, which have the potential to significantly enhance hypothesis generation, especially in large-scale analysis. This approach allows us to uncover intricate interactions, structures, and mechanisms related to the brain and behavior. Once our model is trained, we can use it to classify unseen subjects. Several studies have applied machine learning algorithms to the fMRI data. These algorithms can be used for both classification and prediction. Techniques like logistic regression, k-nearest neighbor, decision tree, and random forest have been utilized to separate MDDs from healthy controls. In a study, a combination of similarity matrices derived from the structural and functional MRI yielded highly accurate classification results (Mousavian et al., 2021). Support Vector Machine (SVM) successfully differentiated between bipolar and MDD patients using features extracted from gray matter (structural MRI) (Rubin-Falcone et al., 2018), fractional anisotropy (diffusion tensor images) (Deng et al., 2018), and spatially independent components (resting-state fMRI) (Gao et al., 2017). This algorithm also classified current MDDs, remitted MDDs, and normal controls, using the Hurst exponent of the resting-state fMRI (Jing et al., 2017). Furthermore, elastic net regression was used as a feature selection technique combined with a Random Forest Classifier (RFC) to distinguish between bipolar and unipolar MDD patients based on task-related fMRI studies. This method effectively classified the two groups by leveraging both neuroimaging and clinical data (Manelis et al., 2020). However, there are limited studies that have examined the MDDs with SI by machine learning approaches (Zheng et al., 2022; Chen et al., 2023; Hasey et al., 2020).

In this study, we have focused on SI as it pertains to mental disorders. To compensate for the effects of small sample sizes, we used decision trees and forests (Zhang et al., 2016; Shaikhina et al., 2019). Additionally, data augmentation techniques have been used to address the inherent limitation posed by sample sizes (Fong et al., 2020; Han et al., 2018; Hagos and Kant, 2019).

To overcome the curse of dimensionality, we used feature selection methods (Chen et al., 2020). There are two essential categories of feature selection techniques: discrimination-based and reliability-based. While discrimination-based feature selection performs better in predictive accuracy, reliability-based feature selection has a higher performance in mapping stability (Xu et al., 2020). Feature selection methods can also be partitioned into three main categories: filter, wrapper, and embedded. The sequential floating forward and backward selection algorithms located within the wrapper category are some of the most robust feature selection algorithms. To achieve accurate and meaningful results, cross-validation and linear discriminant analysis have employed (Stoyanov et al., 2019; Mandelkow et al., 2016; Noirhomme et al., 2014).

The purpose of the current study is to identify SI in treatment-resistant MDD patients using rs-fMRI and to distinguish them from two control groups (treatment-resistant MDD without SI and HCs). We postulate that SI is associated with abnormal brain activations in several critical brain regions.

There is a gap in the application of machine learning to the study of MDD patients with SI. To address this gap, we first extract fALFF, DC, VMHC, and ReHo from the rs-fMRI data. Following this, we apply sequential floating backward selection (SFBS) to identify the most efficient features for differentiating the three subject groups. For classification, we use a permutation test and based on the results, we employ RFC and Elastic Net (EN) in regions demonstrating statistically significant differences.

## 2 Materials and methods

### 2.1 Subjects

All participants in the study (56 females; 16 MDD with SI, 20 MDD without SI, and 20 HC) were carefully screened and evaluated by the Atieh Clinical Neuroscience Center to ensure their psychological suitability. The average age of the participants (all female) for the MDD group with SI, MDD without SI and HC groups were 31 ± 2.13, 32.73 ± 2.56, and 31.34 ± 1.84 years [mean ± Standard Error of the Mean (SEM)] respectively. All participants had a minimum of 8 years of education (from primary school), with average years of education being 14.15 ± 0.65, 13.73 ± 0.73, and 14.10 ± 0.48 years for the respective groups. The inclusion criteria for the study were as follows: (1) female gender; (2) age between 20 to 50 years; (3) a score >7 on the Scale for Suicide Ideation (SSI) for MDD with SI group; and (4) a score exceeding 16 on the Hamilton questionnaire for both MDD with and without SI. The exclusion criteria were: (1) concurrent or historical diagnosis of bipolar disorder, Attention Deficit Hyperactivity Disorder (ADHD), Obsessive-Compulsive Disorder (OCD), schizophrenia, post-traumatic stress disorder, alcohol or substance dependence; (2) recent use of antidepressants (participants were allowed to take common medications as prescribed by their psychiatrist); (3) smoking on the day of the appointment; (4) prior or current treatment with transcranial magnetic stimulation, electroconvulsive therapy, neurofeedback, etc.; (5) pregnancy; (6) claustrophobia; and (7) the presence of metallic materials in the body.

There is no problem when we are blind to drug usage of our all-female subjects because the effect of a specific drug can be different on males and females (We do not know which medicines are being used by the subjects, so considering one sex can be an advantage despite the inherent limitations of this approach). Various studies have demonstrated that women may respond differently to some antidepressants compared to men (Gorman, 2006; Sramek et al., 2022). Consequently, we concentrated our analysis on a group predominantly composed of women (Carr and McKernan, 2015; Weissman et al., 1993). However, this focus has been acknowledged as a limitation in the paper.

The study included a total of 18 female patients diagnosed with major depressive disorder (MDD) and suicidal ideation (SI). Two subjects did not meet the inclusion criteria of the SSI questionnaire and were subsequently excluded. Additionally, three other subjects, one from MDD without SI, and two from the HC group were excluded due to distortions in their MRI images occurring during data acquisition. Before data acquisition, all participants provided written informed consent, and ethical approval was obtained from the Iran University of Medical Sciences.

### 2.2 Hamilton and SSI questionnaire assessments

The Hamilton questionnaire (HDRS) includes 17 items in three parts (Williams, 1988). In the first part, every item has scores between 0 and 4, in the second part, it has scores between 0 and 3, and in the last part, the scores are between 0 and 2. Score 0 means that the related event to the question never happened and the maximum score means that it happens very often. The sum of the scores of these 17 items is between 0 and 53. Higher scores correspond to higher levels of depression severity. Scores between 0 and 7 mean the absence of depression, 8 and 13 mild depression, 14 and 18 moderate depressions, 19 and 22 severe depressions, and higher than 23 very severe depressions.

The SSI questionnaire consists of 19 items. Every item has a score between 0 and 2. The sum of the scores is between 0 and 38. After verification by the psychiatrists and the Hamilton questionnaire, SSI was asked of the participants. Subjects with a total score of less than seven were excluded from the study as they were considered non-suicide ideators. A total score of more than seven means that the patient has a level of suicidal thoughts that can put her life at risk.

### 2.3 Data acquisition

The data were acquired at the NBML using a 3T MRI scanner (Siemens Magnetom Prisma) and a 20-channel receive phase array head coil. Anatomical T1-weighted images were obtained using an MPRAGE sequence and were used as a reference for the registration of the resting-state functional MRI (fMRI) data. T1 image acquisition parameters were: echo time (TE) = 3.45 ms; repetition time (TR) = 1,810 ms; inversion time (TI) = 1,100 ms; flip angle = 7 degrees; a total of 160 sagittal slices with 1 mm resolution (1 × 1 × 1 mm voxel size); and 264 × 264 mm field of view (FOV).

Resting-state fMRI data were acquired using an EPI pulse sequence with TE = 30 ms, TR = 2,000 ms, flip angle = 80 degrees, 35 transverse planes with 4 mm resolution, voxel size = 3 × 3 × 4 mm, 80 × 80 matrix, and 240 × 240 FOV.

### 2.4 Preprocessing

We carried out voxel-based analysis using Data Processing Assistant for Resting-State fMRI (DPARSF) version 5.1 (Yan and Zang, 2010). DPARSF is an rs-fMRI toolbox that encompasses a pipeline for data analysis of rs-fMRI. Initially, we obtained DICOM files for each subject and arranged them in a separate path for functional and anatomical data. We then applied the following preprocessing steps: (a) removed the first 10 volumes due to hemodynamic response delay and instability; (b) corrected slice timing; (c) estimated and corrected head motion parameters during realignment with the first slice; (d) reoriented images by moving the origin to the center of the imaged volume; (e) performed skull stripping using BET; (f) co-registered functional data to anatomical data; (g) carried out segmentation of EPI images with T1 images using affine transformation of the East Asia template onto the control group images; (h) removed motion artifacts by employing nuisance covariates regression using a high order regression model called Friston method with 24 parameters (Due to head motion, one subject from each of the HC and MDD groups, and two subjects from the SSI group were excluded from the study. These subjects had a movement of exceeding 1.5 voxel sizes, horizontally or vertically, or a rotation of more than 3 degrees. The procedure mentioned is a standard preprocessing step in fMRI data analysis. Additionally, we assessed group differences in head movements and as the head movement data did not exhibit a normal distribution, we employed the non-parametric Kruskal-Wallis test to compare the three groups. The results revealed no significant differences in head movement between the groups: *p*-value (rotational movement) = 0.527; *p*-value (horizontal) = 0.541; *p*-value (vertical) = 0.309. (i) performed scrubbing to detect volume images having head motions exceeding the predetermined criteria and regressed them out [we were a little generous in determining the FD threshold for resting-state fMRI data by setting it to 0.55 mm, and we removed only one bad time point before and after spike detection. Finally, we do a Loess smoothing (with the time point removed) and then a cubic interpolation for the missing data point. With these steps, we tried to keep our results away from unwanted data. We also investigated the differences in mean displacement between the three groups. The results are as follows; p-value = 0.386]; (j) removed the effect of non-gray matter tissues like CSF and WM by using their mean values; (k) extracted low-frequency fluctuations and removed biological phenomena like respiratory signals and heartbeats by applying a band-pass filter with a frequency range between 0.01 Hz and 0.1 Hz; (l) normalized the data to the MNI atlas with 3 × 3 × 3 mm resolution using the Diffeomorphic Anatomical Registration Through Exponentiated Lie algebra (DARTEL) template; and (m) increased reliability and SNR by smoothing the data with DARTEL, using a spatial filter with 4 × 4 × 4 mm FWHM.

### 2.5 Voxel-based analysis

#### 2.5.1 Fractional amplitude of low-frequency fluctuations

**Figure 1:**
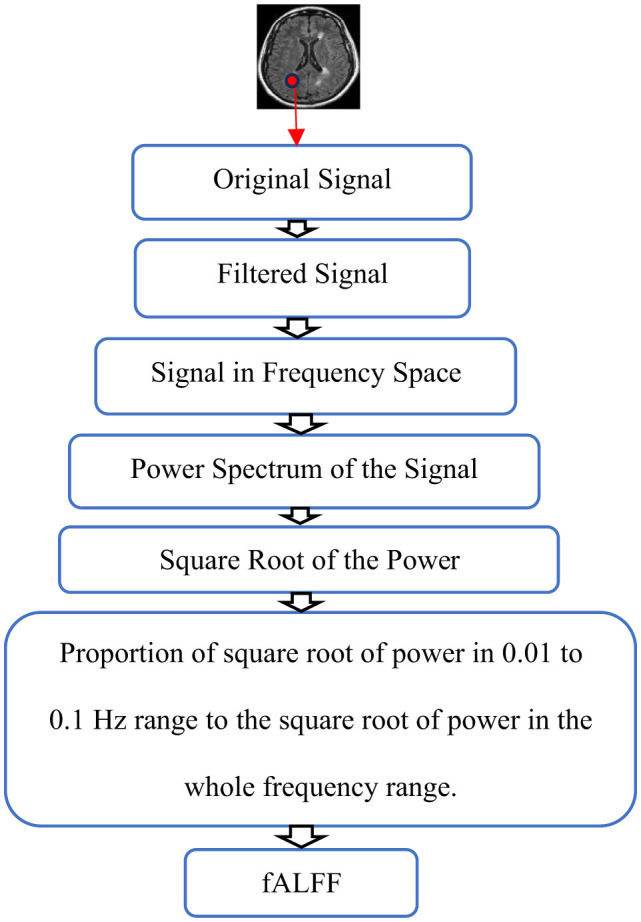
Schematic representation of fALFF calculation. The Original signal is filtered and the power spectrum of the filtered signal is calculated in frequency space. Finally, fALFF is obtained by the ratio of the square root of power in the 0.01 to 0.1 Hz range to the square root of power in the whole frequency range.

To calculate the fractional amplitude of Low-Frequency Fluctuations (fALFF), time courses were transformed to the frequency domain by performing the Fast Fourier Transform (FFT), the power spectrum of the signals was calculated, and, since power is proportional to the square of the amplitude of the time series in the frequency domain, the square root of the power was determined and averaged not only between 0.01 Hz and 0.1 Hz but also in all frequency ranges. After obtaining the square root of the FFT amplitudes in both limited and whole frequency ranges, the ratio of the power of the filtered signals to the power of unfiltered signals was calculated. This ratio is known as fALFF (Yang et al., 2007; Yu-Feng et al., 2007) as shown in Figure 1.

If our signal is defined by Equation 1, ALFF and fALFF can be calculated by Equations 2, 3, respectively. In these equations, N represents the number of data points in the signal.


(1) $$\begin{array}{l} x\left(t\right)=\sum_{i=1}^{N}\left[a_{i}\cos\left(2\pi f_{i}t\right)+b_{i}\sin\left(2\pi f_{i}t\right)\right] \end{array}$$

(2) $$\begin{array}{l} ALFF=\sum_{i:{\int \in \left(0.01,0.1\right)}_{i}}\sqrt{\left[a_{i}^{2}\left(f\right)+b_{i}^{2}\left(f\right)\right]/N} \end{array}$$

(3) $$\begin{array}{l} fALFF=ALFF/\sum_{i:{\int \in \left(0.01,0.25\right)}_{i}}\sqrt{\left[a_{i}^{2}\left(f\right)+b_{i}^{2}\left(f\right)\right]/N} \end{array}$$

The range of the integral is from *f*_min_ = 0.01 to *f*_max_ = 0.1 in Equation 2, and *f*_min_ = 0.01 to *f*_max_ = 0.25 in Equation 3. These formulas will be calculated for i data points in fMRI data.

#### 2.5.2 Regional homogeneity

To assess the Regional Homogeneity (ReHo), we ranked the time series of the fMRI data and examined the correspondence among these ranked signals. Kendall's coefficient of concordance (KCC) was then calculated for each voxel to measure the similarity between its time series and adjacent voxels. The number of adjacent voxels used in this calculation can be either 6,re 18, or 26.


(4) $$\begin{array}{l} W=\frac{\sum {\left(R_{j}\right)}^{2}-n{\left(\bar{R}\right)}^{2}}{\frac{1}{12}K^{2}\left(n^{3}-n\right)} \end{array}$$

In Equation 4, *R*_*j*_ is the sum rank of the jth time point as $R_{j}=\sum_{i=1}^{k}r_{ji}$, where *r*_*ji*_ is the rank of the jth time point of the ith voxel, $\bar{R}=\frac{\left(n+1\right)k}{2}$ is the mean of *R*_*j*_*s*, *K* is the number of time series inside a region, *n*is the number of ranks, and *W*is the KCC among given voxels, which is in the range of 0 to 1 (0 ≤ *W* ≤ 1). In this study, *K* = 27 (one given voxel (center voxel) plus 26 neighboring voxels) and *n* = 221 time points. As a measure of local connectivity of brain areas, ReHo is calculated by measuring the homogeneity of time series among brain voxels and the low-frequency content of the fMRI time series. The KCC of the center voxel is calculated using its neighboring voxels. This process was repeated for all voxels in the brain, generating a ReHo map for each subject in both groups (Zang et al., 2004; Wu et al., 2009; Al-Zubaidi et al., 2019).

#### 2.5.3 Degree centrality

Degree Centrality (DC) is a method for measuring whole-brain connectivity. This method first calculates Pearson's correlations between the time series of a voxel and all other voxels in the brain. A threshold of 0.25 is applied to these correlations before calculating DC (Gao et al., 2016; Al-Zubaidi et al., 2019).

Equation 5 shows the *N*×*N* Pearson's correlation coefficient matrix.


(5) $$\begin{array}{l} Correlation\;Matrix\;=\;\left[\begin{array}{ccc} r_{11} & ... & r_{1j}\\ \vdots & \ddots & \vdots \\ r_{i1} & ... & r_{ij} \end{array}\right] \end{array}$$

(6) $$cd_{ij}=\{\begin{array}{cc} 0 & r_{ij}<0.25\\ 1 & r_{ij}\geq 0.25 \end{array}$$

(7) $$\begin{array}{l} CD_{i}=\sum_{j=1}^{N}cd_{ij} \end{array}$$

For binarizing purposes, the value of 0 is assigned to cd_ij when r_ij < 0.25 and 1 when r_ij≥0.25. Finally, the DC of each voxel in the brain is obtained by summing all significant correlations as illustrated in Equation 7. Notably, the importance of a voxel (node) is determined by the number of voxels that are correlated with it.

#### 2.5.4 Voxel-mirrored homotopic connectivity

Voxel-mirrored Homotopic Connectivity (VMHC) is a voxel-wise measurement that calculates the connectivity between the hemispheres (Su et al., 2016). This method plays a crucial role in fMRI studies of neurological disorders such as schizophrenia (Hoptman et al., 2012; Woon et al., 2010; Guo et al., 2018), depression (Guo et al., 2013), somatization disorder (Su et al., 2016), and Parkinson's disease (Su et al., 2016). It demonstrates abnormal homotopic connectivity and synchronization of spontaneous activity patterns between co-located regions of the two hemispheres. This feature of the functional structure of the brain is very important for understanding brain function (Wei et al., 2018).

### 2.6 Data augmentation

**Figure 2:**
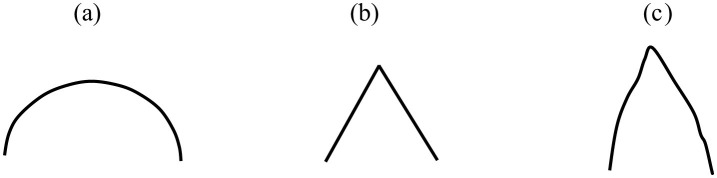
**(A)** An example of a spline. **(B)** A representation of a fake fMRI signal, **(C)** multiplication of **(A, B)**.

After preprocessing the data, magnitude warping (Um et al., 2017) was performed on the time series of 116 AAL Atlas regions. In this procedure, the magnitude of each time series is multiplied by a curve generated through a cubic spline with four knots, and random magnitudes with a mean of 1 and a standard deviation of 0.2 are assigned to these knots. Figure 2A illustrates an example of a spline. Suppose this cubic curve is multiplied by an fMRI signal, a portion of which is depicted in Figure 2B. After this multiplication, the fMRI signal will experience slight smoothing and a minor change in magnitude. Figure 2C displays the result of this process (note that these figures are for illustrative purposes only). This operation generates a new signal with characteristics similar to the original, which can aid our machine learning algorithm in generalizing better and improving classification accuracy by exposing it to a more varied dataset (Iwana and Uchida, 2021). To prevent information leakage from the training data to the test data, at the very beginning, eight subjects (2 MDDs with SI, 3 MDDs without SI, and 3 HCs) were designated as test data. Additionally, another eight samples were selected as validation sets. These samples were used to monitor model performance and prevent overfitting. Therefore, we had 40 patients in our training, which was then augmented to produce an additional dataset of the same size. This resulted in 80 subjects for the study, comprising 28 HC, 28 MDD without SI, and 24 MDD with SI.

### 2.7 Feature selection and classification

We obtained four feature maps indicating brain activation by calculating fALFF, ReHo, DC, and VMHC from the fMRI data. These maps were masked using the Automated Anatomical Atlas (AAL) (Tzourio-Mazoyer et al., 2002), which consists of 116 regions of interest. The mean value of each region was averaged, producing a 1D numerical array for each map. We collated these vectors for all subjects to form a 2D matrix, where the first dimension represented the number of individuals (16 MDDs with suicide ideation, 20 MDDs without suicide ideation, and 20 HCs), and the second dimension represented the number of features (ROIs). To mitigate overfitting as much as possible, we applied feature selection to the training set before data augmentation. We separated the test (8 individuals) and validation (8 individuals) datasets from the beginning. The remaining 40 subjects were used for training, and feature selection was applied to this training set. The selected features were applied to all augmented, test, and validation datasets in subsequent steps. To select features, we utilized the Sequential Floating Backward Search (SFBS) method. This method starts with the complete set of features and iteratively eliminates less important ones to acquire the optimal subset of a predetermined size while retaining the most significant ones. The algorithm operates in two stages: first, it excludes features to enhance performance. Second, it conditionally includes an excluded feature if it improves classifier performance (Chowdhury and Turin, 2020). The SFBS algorithm's working mechanism is as follows - for an input *X* = {*x*_1_, *x*_2_, ..., *x*_*n*_} (where n = 80 in this study), the SFBS algorithm feeds all features as input and generates an output *Y*_*k*_ = {*y*_*i*_|*i* = 1, 2, ..., *k*; *y*_*i*_∈*X*} where *k*<*n*. This process involves two stages: features exclusion and conditional feature inclusion. First, a feature is eliminated from the feature subset to improve performance. Second, the excluded feature is added back if it enhances classifier performance. The threshold for feature selection was determined based on data correlation. For uncorrelated data, N-1 features were considered, while for highly correlated data. $\sqrt{N}$ features were used. Here N represents the number of samples in the study (Hua et al., 2005).

**Figure 3:**
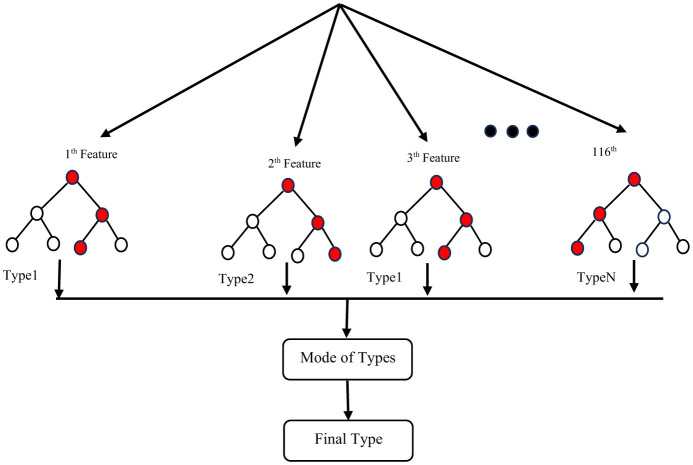
Implementation of an RCF.

To address the issue of small sample size and improve the generalizability of the trained model, we performed feature reduction. This involved eliminating less significant features. Feature reduction offers several benefits: faster training, reduced model complexity, improved model accuracy, and lower overfitting. For classification, as mentioned before, we chose a linear method (Elastic Net) and a non-linear method (Random Forest Classifier) to investigate their impact on the final results. After selecting the most important features, we used nested k-fold (k = 5) cross-validation with a grid search algorithm on the training set to identify optimal hyperparameters for both the Random Forest Classifier (RFC) and Elastic Net (EN). These hyperparameters included the number of trees in the forest (n-estimators), the maximum number of features to consider when searching for the best split (max-features), a constant that multiplies the penalty terms (α), and the Elastic Net mixing parameter (l1-ratio) for EN. After evaluating a range of reasonable values for these parameters, we found that the optimal parameters for the best estimator were n-estimators = 200, max-features = 2, α = 0.01, and l1-ratio = 0.9. Training accuracy for different hyperparameter values is shown in Table 1, and Table 2 for ElasticNet and RFC respectively. Setting max-features to 2 automatically triggers the code to run with a vector containing three parameters: [“auto”, “sqrt”, “0.2”]. The best accuracy with these parameters will be reported. After completing feature selection hyperparameter optimization, we trained our model using the original training data. The results were not as satisfying as we expected, so we applied data augmentation to the training data. We trained our model using augmented data to improve the classification accuracy. For classification, we employed an RFC, a supervised learning algorithm consisting of multiple decision trees trained independently with different samples. The RFC algorithm aggregates the predictions of these trees to make a prediction. The RFC algorithm selects random samples from the dataset, builds a decision tree for each selected sample, aggregates the prediction of this tree by voting (selecting the most frequent prediction). Finally, the algorithm determines the voted prediction as the final prediction. Figure 3 provides a visual representation of the RFC implementation.

**Table 1:** Elastic net; mean training accuracy for different hyperparameter values.

**l1-ratio**	0.1	0.4	0.6	0.9
**α**	0.001	0.001	0.001	0.001
**Accuracy**	0.925	0.0.928	0.961	0.988
**α**	0.01	0.01	0.01	0.01
**Accuracy**	0.942	0.952	0.990	0.100
**α**	0.1	0.1	0.1	0.1
**Accuracy**	0.933	0.945	0.982	0.987
**α**	0.5	0.5	0.5	0.5
**Accuracy**	0.902	0.916	0.976	0.953

**Table 2:** RFC; mean training accuracy for different hyperparameter values.

**Max_features**	**“Auto”**	**“Sqrt”**	**0.2 (20%)**
n_estimators	50	50	50
Accuracy	0.967	0.962	0.966
n_estimators	100	100	100
Accuracy	0.986	0.981	0.986
n_estimators	200	200	200
Accuracy	0.100	0.999	0.100
n_estimators	500	500	500
Accuracy	0.100	0.999	0.100

Dataset [80 participants from a total of 96 (16 samples separated for test and validation from the original data)].

In addition to the RFC, we employed the EN as an embedded method. The EN combines the advantages of both wrapper models, which interact with the classification model, and filter models, which are less computationally intensive than wrapper models. This regularized regression method linearly combines *L*_1_ and *L*_2_ penalties, drawn from the LASSO and Ridge algorithms during training. We used the penalty parameters obtained through feature selection to select the most important features. The overlapping features between Sequential Floating Backward Search (SFBS) and Elastic Net were fed into a Python implementation of an EN classifier to distinguish between the three groups.

The ruling formula for this algorithm is illustrated in Equation 8 (Zou and Hastie, 2005).


(8) $$\begin{array}{c} \hat{\theta }=argmin_{\theta }\left(||y-X\theta |{|}^{2}+\lambda_{2}||\theta |{|}^{2}+\lambda_{1}||\theta |{|}_{1}\right) \end{array}$$

where


(9) $$\begin{array}{c} ||\theta |{|}_{1}=\sum_{i=1}^{p}|\theta_{i}|\text{and}||\theta |{|}^{2}=\sum_{i=\;1}^{p}{\theta_{i}}^{2} \end{array}$$

To have the best model we need to choose best weights that makes our predicted data with its coefficients similar to the observed data as much as possible. So according to the formula (Equation 8) we try to minimize loss function Ô by making Residual Sum of Squares (||*y*−*Xθ*||^2^), L_2_ Penalty ($\lambda_{2}||\theta |{|}^{2}$) and L_1_ Penalty (λ_1_||θ||_1_) as small as possible.

Considering α as $\frac{\lambda_{2}}{\lambda_{1}+\lambda_{2}}$, and λ_1_ as l_1__ratio we will have the following formula which applicable in python:


(10) $$\begin{array}{l} \hat{\theta }=\;argmin_{\theta }({\left\|y-X\theta \right\|}^{2}+\alpha .[(1-l_{1}\_ratio)]{\left\|\theta \right\|}^{2}\\ \;+l_{1}\_ratio{\left\|\theta \right\|}_{1}) \end{array}$$

Solving Equation 8 will solve the optimization problem, and the total Elastic Net penalty would be:


(11) $$\begin{array}{c} ElasticNet_{Penalty}=\left({\alpha L_{1}}_{Penalty}+\left(1-\alpha \right){L_{2}}_{Penalty}\right) \end{array}$$

**Figure 4:**
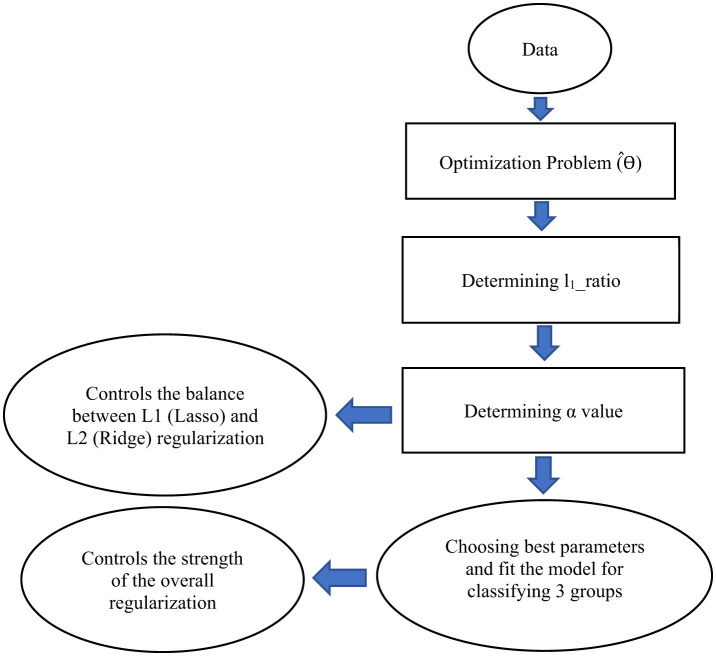
A schematic illustrating the steps to develop an Elastic Net (EN) model for classifying three groups. This schematic represents only the architecture of the EN framework. In our study, we combine Sequential Feature Backward Selection (SFBS) with EN to identify the optimal set of features for classification. EN has been implemented as a classifier not just as a regularization model. For this purpose, it can be implemented as a LogisticRegression with the option penalty = “elasticnet” in python.

Figure 4 shows a schematic for steps showing how an EN is applicable on a dataset.

Lastly, to estimate the classification accuracy, we calculated the error rate. We permuted the selected features randomly 10,000 times to obtain an empirical distribution of the average difference (*p* < 0.05).

**Figure 5:**
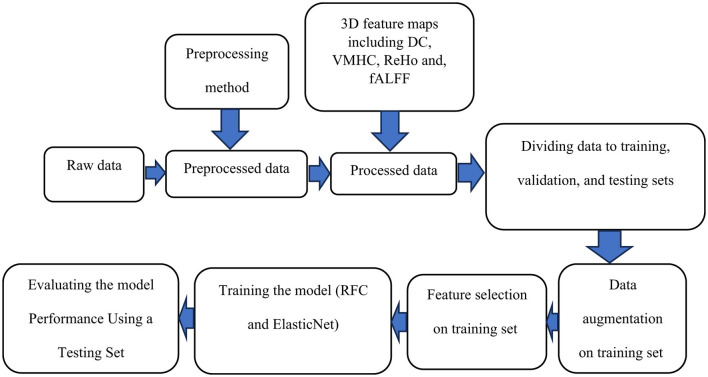
A schematic outlining the steps for data preparation and processing is shown. Initially, raw data undergoes preprocessing. The preprocessed data is then used to compute voxel-based connectivity measures, which serve as features for training. To enhance the model, data augmentation is applied to the training data, followed by feature selection. The model is trained using these features and evaluated with a test dataset. Finally, the results are reported.

Figure 5 shows a general schematic outlining the steps for data preparation leading to the final results.

## 3 Results

Table 3 shows the demographic, clinical, and behavioral characteristics of the three groups (MDDs with suicide ideation, MDDs without suicide ideation, and healthy controls). The suicide ideators, non-suicide ideators, and HCs were well-matched in terms of age and education level. There was no significant difference in HDRS scores between MDDs with and without SI.

**Table 3:** Clinical demographics of SIs, NSIs, and HCs.

**Variables (Mean ±SEM)**	**SIs**	**NSIs**	**HC**	***P*-value, FDR corrected**
Gender (female)	16	20	20	—
Age (years, from 20 to 50)	31 ± 2.13	32.73 ± 2.56	31.34 ± 1.84	0.383^a^
Education level (years, from 8 to 16)	14.15 ± 0.65	13.73 ± 0.73	14.10 ± 0.48	0.116^a^
HDRS	23.13 ± 2.14	21.77 ± 1.91	—	0.098^b^
SSI	19.06 ± 1.66	—	—	—

SI, Suicide Ideators; NSI, Non-Suicide Ideators; SEM, Standard Error of Mean; HDRS, Hamilton Depression Rating Scale; SSI, Scale for Suicide Ideation.

^a^Using ANOVA; ^b^Using two sample t-test.

For each 3D map generated from the fALFF, ReHo, DC, and VMHC analyses, we selected a maximum of seven features [representing different brain regions according to the AAL atlas (Tzourio-Mazoyer et al., 2002)] for classification, based on the guidelines outlined in Al-Zubaidi et al. (2019). We monitored accuracy during training and selected the optimal number of features by setting the maximum to seven. Among the four maps, fALFF resulted in the highest classification accuracy, achieving 92.50% and 87.50% for RFC and EN, respectively. We obtained confusion matrices from the classification results for all four maps and calculated performance metrics. Since we had a classification problem for three groups, the confusion matrix consisted of nine elements (as shown in Table 4). The overall confusion matrix is calculated by averaging the five different confusion matrixes across the folds. Using this matrix, we calculated accuracy, misclassification, sensitivity, recall, specificity, precision, and F1 Score using the following formulas: Accuracy = (TP + TN)/All Predictions, Misclassification = 1 - Accuracy, Sensitivity = Recall = TP/(FN + TP), Specificity = TN/(TN + FP), Precision = TP/(TP + FP), and F1 Score = 2 × (Precision × Sensitivity)/(Precision + Sensitivity). These values are presented in Table 4.

**Table 4:** RFC and EN classification results.

**fALFF**	**Confusion matrix**	**Accuracy**	**Misclassification**	**Sensitivity**	**Specificity**	**Precision**	**F1-Score**
RFC	$\left[\begin{array}{ccc} 1.6 & 0.4 & 0\\ 0.4 & 2.2 & 0.4\\ 0 & 0.4 & 2.6 \end{array}\right]$	0.9000	0.1000	0.8000	0.9333	0.8000	0.8000
EN	$\left[\begin{array}{ccc} 1.4 & 0.6 & 0\\ 0.4 & 2.2 & 0.4\\ 0 & 0.4 & 2.6 \end{array}\right]$	0.8750	0.1250	0.7000	0.9333	0.7778	0.7369

Average confusion matrix and evaluation scales were obtained using features selected from the fALFF map (positive class: MDD with SI, Negative classes: MDD without SI and HCs).

TP (True Positive): The subject is really SI and we predicted it right. The actual value and predicted value should be the same. So concerning the SI class, the value of Cell 1 is the TP value.

FN (False Negative): The subject is actually SI, but we classified her as MDD or HC. The sum of values of corresponding rows except for the TP value.


$$\begin{array}{c} \text{FN}=\left(\text{Cell }2+\text{Cell }3\right) \end{array}$$

FP (False Positive): The subject is not SI, but we consider her as one. The sum of values of the corresponding column except for the TP value.


$$\begin{array}{c} \text{FP}=\left(\text{Cell }4+\text{Cell }7\right) \end{array}$$

TN (True Negative): The subject is not SI and we predicted it right. The sum of values of all columns and rows except the values of that class that we are calculating the values for.


$$\begin{array}{c} \text{TN}=\left(\text{Cell }5+\text{Cell }6+\text{Cell }8+\text{Cell }9\right) \end{array}$$

We see that just TP includes one cell, and all other three parameters contain the summation of at least 2 cells. So determining a TP value can be challenging if we not choose or fit our model in the best way.

To have a better understanding of the model and its limitations, there is some details of how we calculate TP, FN, FP, TN in Table 5. When dealing with three classes the confusion matrix is similar to that of two classes, although the calculation is a little different. We considered SI as a reference and compared this group with other groups (MDD without SI and HC).

**Table 5:** A general example of confusion matrix calculation for a three-category classification problem.

	**Predicted values**			
**Actual values**		**SI**	**MDD**	**HC**
	SI	Cell 1	Cell 2	Cell 3
	MDD	Cell 4	Cell 5	Cell 6
	HC	Cell 7	Cell 8	Cell 9

Permutation testing revealed that, only 3 out of 6 selected features fALFF (Frontal_Sup_L, Frontal_Sup_Orb_R, and Cingulum_Mid_R) showed statistically significant differences between individuals with MDD, with and without suicidal ideation. Four brain regions (Frontal_Sup_R, Frontal_Sup_L, Cingulum_Ant_L, and Hippocampus_L) were identified as important for classifying MDDs without suicide and HCs. Five regions (Frontal_Sup_Orb_R, Frontal_Sup_R, Frontal_Sup_L, Cingulum_Mid_R, and Hippocampus_L) were identified as important for classifying MDDs with SI, and HCs.

The above studies and evaluations were also done for the other three connectivity maps. The DC map resulted in 86.00% accuracy for RFC and 87.12% for EN. The ReHo map showed an accuracy of 80.33% for RFC and 76.20% for EN. The VMHC map resulted in 85.60% accuracy for RFC and 85.16% for EN.

The brain regions identified as important features after permutation testing for VMHC, DC and ReHo, respectively were; [Frontal_Sup_L Frontal_Sup_R, Frontal_Sup_Orb_R, Precentral_L**]** (VMHC), [Frontal_Sup_L, Frontal_Sup_R, Cingulum_Ant_L, Cingulum_Mid_R, Precentral_L] (DC), [Frontal_Sup_L, Frontal_Mid_L] (ReHo) respectively.

**Figure 6:**
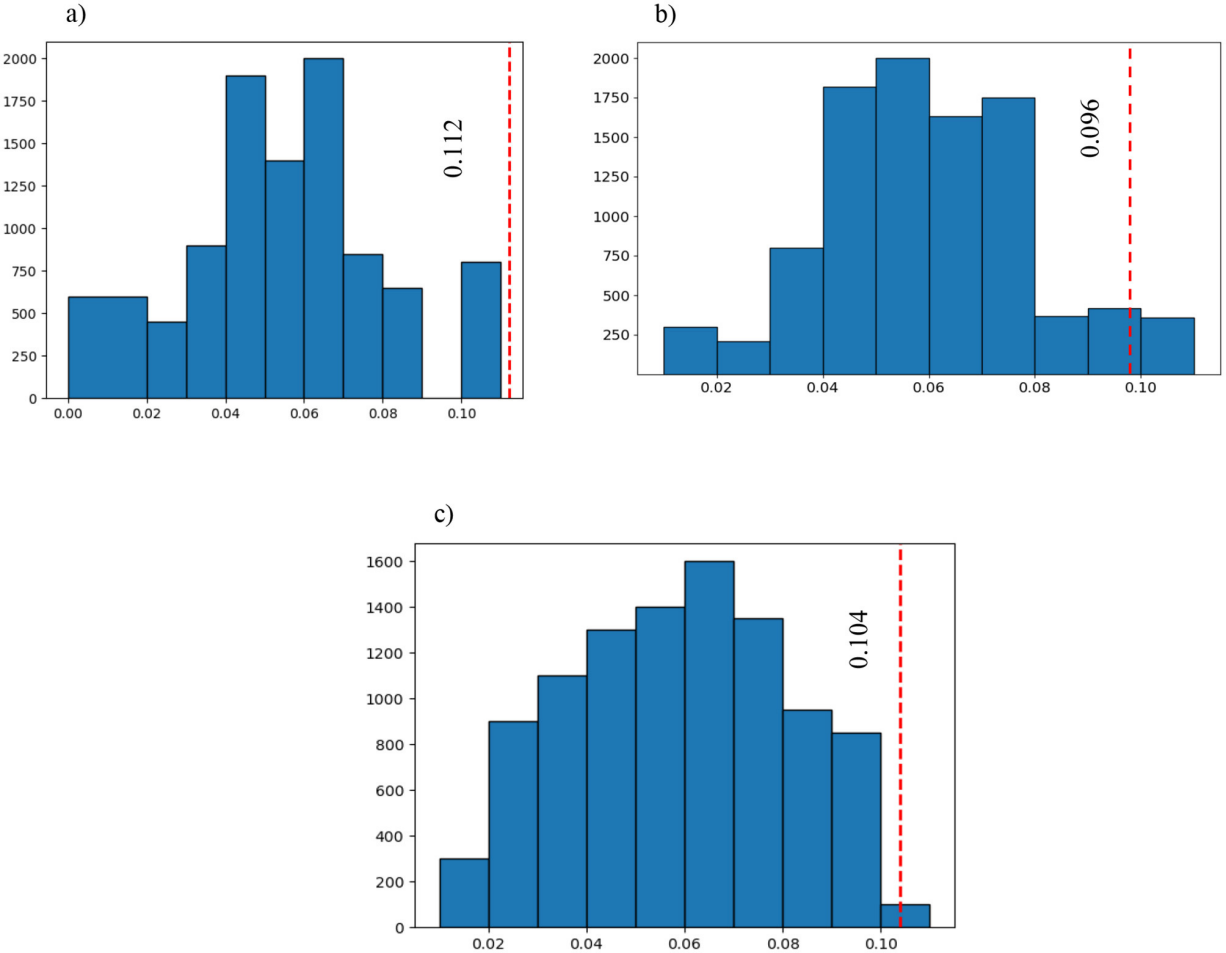
The blue graph represents all the permuted differences for the 3 of the 6 selected features that were significantly different between the three groups (large bin size has been chosen to have a better representation): **(A)** the first selected feature after SFBS and the permutation test (*p* = 0.00001); **(B)** the sixth selected feature after SFBS and the permutation test (*p* = 0.04239); and **(C)** the third selected feature after SFBS and the permutation test (*p* = 0.00241). The red vertical line is the ground truth value which shows the difference between the selected features in the three groups before permutation.

Figure 6 illustrates the outcomes of the permutation test performed on the chosen features. Subsequently, after selecting the most significant features, the absolute difference in average values was calculated for each feature separately and labeled as the Ground Truth (GT) score. The vectors of features for both groups underwent permutation 10,000 times to obtain permuted features, and the absolute difference in mean values was computed for each permutation and compared to the GT. If the *p* < 0.05, it indicates that the majority of absolute differences observed during permutation were smaller than the original GT.

Our research has made a significant discovery in which the brain regions associated with the default mode network (DMN) and central executive network (ECN) were consistently implicated across all connectivity maps. This finding shed light on the critical involvement of these two networks in the development of suicidal ideation in women with MDD. Prompt treatment targeting these networks may help reduce the severity of suicidal thoughts.

**Figure 7:**
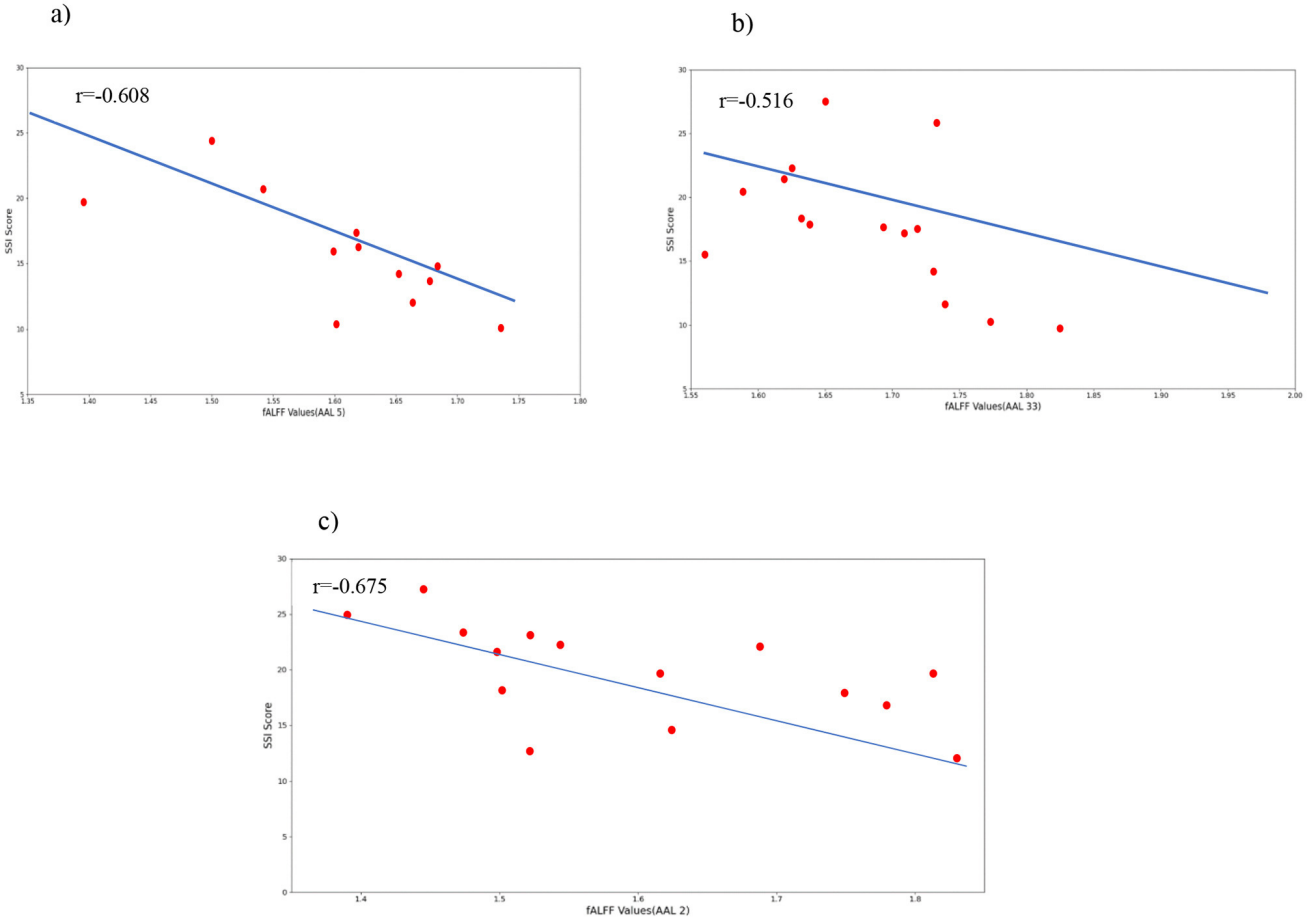
Correlations between selected fALFF values and the clinical behavior scale related to suicide ideation (SSI). **(A)** correlation between Frontal_Sup_Orb and SSI scores; **(B)** correlation between Cingulum_Mid and SSI scores; **(C)** correlation between Frontal_Sup_L and SSI scores.

**Figure 8:**
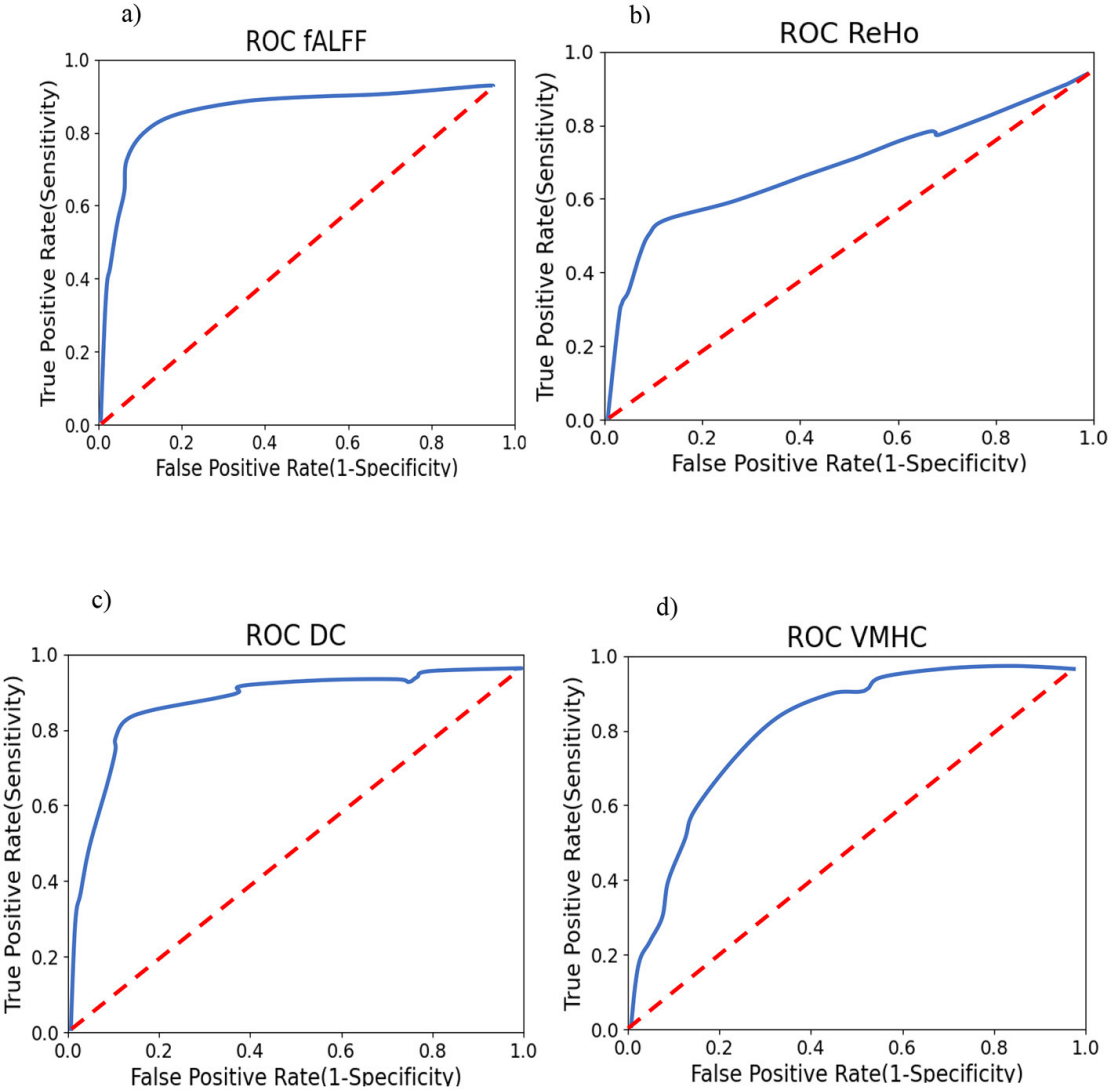
Smoothed One-vs-Rest (suicide ideators vs. rest) ROC curves for RFC. Training **(A)** using fALFF features; **(B)** using ReHo features; **(C)** Using DC features; and **(D)** using VMHC features.

To support the results mentioned after the permutation test, we calculated the correlation between SSI scores and mean fALFF values. The fALFF values in Frontal_Sup_L, Frontal_Sup_Orb_R, and Cingulum_Mid_R were negatively correlated with SSI scores (r = −0.675, *p* = 0.004 for AAL 2, r = −0.608, p = 0.013 for AAL 5, and r = −0.516, *p* = 0.041 for AAL 33). Figure 7 shows the results of this correlation analysis, which was conducted to investigate the relationship between average fALFF values and SSI scores. In this study, the final stage of comparing classification performance is to generate receiver operating characteristic (ROC) curves for all connectivity maps. An example of this evaluation method can be seen in Figure 8. To evaluate the efficiency of our proposed model against several existing models, we prepared Table 6. Table 6 shows a comparison between different classifiers including RCF and ElasticNet (EN), a Support Vector Machine (SVM), and a simple Convolutional Neural Network (CNN). Results showed that RFC outperformed others, while the CNN-based exhibited the lowest accuracy. This may be attributed to the complexity of CNN and the overfitting of the model caused by an inadequate sample size for the CNN model.

**Table 6:** A comparison between the models used in this study and two other methods.

**Method**	**CNN**	**SVM**	**ElasticNet**	**RFC**
Accuracy (%)	74.0%	87.5%	87.5%	90.0%

## 4 Discussion

This study utilized rs-fMRI to distinguish between MDD female patients with and without clinical suicidal ideation and HCs. The participants in the study exhibited varying degrees of suicidal ideation, ranging from occasional ideation to active planning and determination to carry out an act. Connectivity maps derived from fMRI data were used to classify the groups, employing state-of-the-art feature selection and classification methods. Following feature selection (using SFBS), permutation testing, and RFC as a powerful classifier, significant features of all connectivity maps were identified, including Frontal_Sup and Frontal_Sup_Orb (belong to DMN) and Cingulum_Mid (belong to central ECN), and were determined to be important regions that distinguish between Suicide Ideators (SIs) and non-Suicide Ideators (NSIs). Additionally, activation scores in these regions were negatively correlated with SSI scores, indicating that a reduction in connectivity measures in these nodes may signal an increase in the severity of suicidal ideation and could lead to active suicide attempts. Therefore, prompt treatment targeted at the responsible brain regions is essential to prevent the development of suicidal ideation, which is the primary factor leading to suicide attempts in most MDD patients.

Some studies examined voxel-based connectivity methods examined in this study. A study investigated the ReHo of three groups: MDD patients with treatment-refractory disease (TRD), non-treatment refractory depression, and healthy controls. ReHo was significantly higher in both depressed groups compared to the control group. Furthermore, the TRD group showed additional cerebral regions compared to the non-TRD group (Wu et al., 2011).

Examining the effects of Electroconvulsive Therapy (ECT) on brain function in adolescents with MDD and SI, and the changes induced by ECT as a treatment mechanism for MDD with SI as a hypothesis, significant changes were found in DC values in the inferior frontal gyrus and left hippocampus, showing reductions and increases, respectively (Li et al., 2021). In a study of female MDD patients, fALFF and ALFF were evaluated. Then, depressed and remitted MDD patients were compared to Healthy Controls (HCs). With ALFF values under consideration, a significant difference was found between remitted MDDs and normal controls in the right precuneus. Additionally, with fALFF measurements, marked differences were seen between current MDDs and healthy controls in the right putamen (Jing et al., 2013). Voxel-Mirrored Homotopic Connectivity (VMHC), a more recent method, for calculating inter-hemispheric functional coordination in several regions was used to investigate connectivity templates between two brain hemispheres in drug-free MDD patients and an HC group. Decreases in VMHC values were observed in the right and left posterior cingulate cortex (PCC) (Fan et al., 2018). One critical issue in classification is selecting the best strategy for feature selection. In practical problems involving data representation, many features may be used, but only a few of them are important and relevant to the study's goal (Kira and Rendell, 1992). Most real-world classification problems require supervised learning, where the class probabilities and class-conditional probabilities are uncertain, and each instance is associated with a class label (Dash and Liu, 1997). In feature selection methods such as filter models, the feature selection stage can be independent of the learning process or iteratively assess the value of chosen features based on the learning algorithm's performance, such as in wrapper models. In our study, we utilized wrapper models by selecting features and prompting a classifier for prediction (Tang et al., 1997). Wrapper models use a specific classifier to evaluate the quality of selected features and provide a simple and robust approach to address feature selection, regardless of the learning machine used (Kohavi and John, 1997). By implementing high-order functional connectivity, Sequential forward selection (SFS) in combination with a sparse regression strategy was performed on fMRI data maps to make the classification between mild cognitive impairment patients and normal controls possible with high accuracy of 84.85% (Chen et al., 2017). Sequential backward selection (SBS) deals with a large number of features excellently (Ladha, 2011). According to Wah et al. (2018), after comparing correlation-based and information gain feature selection algorithms as filter approaches and sequential forward and backward elimination as wrapper methods, the wrapper procedures had better performance in selecting appropriate features in both surrogate and real datasets. Furthermore, SFBS had a slightly better function when it came to a small sample size. Random forest is a combination of sequence of tree-based classifiers. RFC and SBS have been utilized for breast cancer detection and prognostic. Compared to other methods like statistical analysis of mammographic features combined with SVM (93.73%), a fuzzy-based data transformation for feature extrication (96.35%), SVM and evolutionary algorithms (97%), this procedure had a higher classification accuracy (99.82%) on Wisconsin breast cancer diagnosis dataset (Nguyen et al., 2013). In Azar et al. (2014), a genetic algorithm was used for feature selection. Then, the features selected from lymph diseases dataset were fed into RFC. This procedure was able to classify the intended data with 92.2% accuracy. In this study, SFBS outperformed SFFS in combination with RFC. According to Masetic and Subasi (2016), RFC was capable of detecting cognitive heart failure with 100% accuracy, better compared to other classifiers like; C4.5 decision tree, k-nearest neighbor, SVM, and artificial neural networks. Six different classifiers including elastic net, RFC, neural network with one hidden layer, SVM, logitboost, and decision tree were compared to observe their power in discriminating twelve different medical datasets. Here, RFC and elastic net had superior performance (Deist et al., 2018). A hypergraph elastic net had the best clustering and classification ability compared to seven other algorithms: G-graph, LE-graph, l1-graph, KNN-hypergraph, semantic correlation hypergraph, sparse subspace clustering, and low rank representation on three different databases (Liu et al., 2016).

As mentioned in the previous studies selecting determinative features is an important part of classification problems on different data types like fMRI, that is why we chose SFBS and Elastic Net penalty as feature selection procedures and utilized RFC and Elastic Net to generate a remarkable power in separating two proposed groups of study. The central executive network (ECN) is responsible for maintaining and controlling information in working memory, decision-making, and problem-solving. It is highly active during cognitively and emotionally demanding tasks and plays a crucial role in emotion regulation during task-oriented processing. Given these functions, it is not surprising that the ECN is involved in major depressive disorder (MDD) and other cognitive disorders (Menon, 2011). In contrast, the default mode network (DMN) shows strong low-frequency fluctuations during passive tasks that require internal mental-state processing, such as remembering, self-referential processing, autobiographical information processing, imagining the future, and thinking about others (Buckner, 2022; Broyd et al., 2009). Dysfunction of the DMN has been linked to rumination and self-preoccupation, which are common symptoms in patients with MDD (Broyd et al., 2009). Differences in functional connectivity values were observed between various networks, including DMN, ECN, and SN, in resting-state fMRI data from MDD patients with suicidal ideation (SI) compared to healthy controls (HCs) (Fattahi et al., 2021). In young MDD patients with and without a history of suicidal behavior, functional connectivity was assessed between the anterior cingulate cortex (ACC) subregions (which belong to the salience network) and other brain areas. Results showed significant differences in subregions of the ACC-superior frontal gyrus between the two groups (Qiu et al., 2020). Another study used voxel-based morphometric analysis to distinguish gray matter volume differences in the prefrontal cortex (PFC), an area associated with the DMN, between individuals with MDD and SI, subjects with MDD but without suicidal thoughts, and normal controls. The study found reduced gray matter volume specifically in the left and right dorsolateral PFC (DLPFC) and right ventrolateral PFC (VLPFC) in the MDD with SI group compared to the HC and MDD without SI groups (Zhang et al., 2020). A study utilizing the sliding-window analysis to calculate the dynamic amplitude of low-frequency fluctuations (dALFF), a proxy for intrinsic brain activity, in individuals with MDD with and without SI as well as normal controls, showed differences in brain dynamics within several regions belonging to the DMN, including the dorsal anterior cingulate cortex (ACG), the left orbital frontal cortex (ORBsup.L), and the left inferior temporal gyrus (ITG.L), as well as the left hippocampus (HIP.L), which is part of the dorsal attention network (Li et al., 2019). Moreover, voxel-wise whole-brain functional connectivity maps were created, and graph-theoretical-based functional connectivity maps were calculated, revealing meaningful differences in functional connectivity strength between suicide attempters and non-attempters in the right orbitofrontal cortex and bilateral dorsomedial prefrontal cortex, both of which belong to the DMN (Chen et al., 2021). Further analyses using network-based statistics and graph-theoretical approaches revealed that MDD patients with suicidal thoughts displayed different correlation values compared to those without SI and normal controls in the superior frontal gyrus (orbital part) and thalamus, suggesting potential issues with decision-making and information integration (Kim et al., 2017).

In a study that investigated MDD without SI, MDD with SI but without SA, and MDD with SA, it was found that widespread functional connectivity attenuation occurred in MDD patients in both the strongly and weakly connected states, involving the intra-network and inter-network connectivity of the primary networks (VIS, AUD, SMN) as well as the high-level cognitive network (DMN) using SVM. The highest accuracy value of this study was 86.84% (Xu et al., 2022).

In another study that perused the MDDs with SA and HCs, showed that compared HCs, adolescent SAs exhibited reduced ALFF values in the bilateral medial superior frontal gyrus (mSFG) and bilateral precuneus which both located at DMN. These decreased ALFF values were negatively correlated with Child Depression Inventory (CDI) scores, while reduced ALFF values in the bilateral precuneus were also negatively correlated with Suicidal Ideation Questionnaire-Junior (SIQ-JR) scores. Support vector machine (SVM) analyses demonstrated diagnostic accuracy rates of 76.8% for reduced ALFF values in the bilateral mSFG and those in the bilateral precuneus (Zhou et al., 2023).

From a neuropsychological perspective, DMN dysfunction is closely related to psychiatric disorders characterized by emotion dysregulation, such as MDD. The brain regions responsible for social regulation, which is considered crucial for emotion processing and regulation, overlap highly with the DMN (Xie et al., 2016). Moreover, the frontoparietal network and the DMN have been found to play an important role in emotion regulation (Pan et al., 2018). A remarkable association between emotion dysregulation and suicidal ideation was found in adolescents of both sexes in the past year. When lifetime suicide attempt was examined as an outcome factor, SI remained the only factor strikingly associated with it. In addition, examination of both prior and *post hoc* analyzes revealed that limited access to adaptive emotion regulation procedures and the presence of a mood disorder was related to suicidal ideation in the past year (Hatkevich et al., 2019). In another study, affiliation, and emotion regulation approaches, particularly internal dysfunction, showed a strong and moderate association, respectively, with suicidal ideation (Swee et al., 2020). It has also been shown that after applying the Bayesian model to fMRI data, self- and other-referential processing inhibits the flow of information from the left inferior frontal gyrus to some DMN areas in a direct pathway, and this process is consistent with independent inhibition (Soch et al., 2017). The frontoparietal network showed a key function in inhibition, attention, and response control in two experiments based on a go/no-go task (Dodds et al., 2011). On the other hand, cognitive inhibition plays an important role in emotion regulation and adaptation of thoughts and actions (Joormann and Gotlib, 2008). The lack of cognitive inhibition may lead to further suicidal ideation resulting from a lack of regulation of the aroused emotional state and the inspiration of a ruminative and obsessive way of thinking. The suicidal act may be the inevitable consequence of this function in the brain (Richard-Devantoy et al., 2012). In addition, executive dysfunctions such as deficits in cognitive inhibition are common in depressed patients and may be an important factor in suicidality. Indeed, deficits in cognitive inhibition are closely related to suicidal behavior in patients with affective disorders (Richard-Devantoy et al., 2015). Suicidal individuals focus more on negative information, which can be considered an inhibition deficit in these patients. Considering all this, inhibition and emotion regulation, which are important features of risky behavior, are simultaneously linked to DMN and ECN in patients who attempt suicide, in addition to SI.

Machine learning and artificial intelligence (AI) have extended to neurology in recent years. These methods are essential in the creation, management, and preservation of clinical and experimental data. Neuroscience, on the other hand, provides valuable insights into how neural processes occur, which can inform and improve AI algorithms. According to our purpose in the study and the dataset we have, the best method should be selected to get the best results (Mofatteh, 2021; Schuman et al., 2022).

Other populations, like males, people with coexisting conditions, and individuals with cancer, can be future groups of study. Existing brain tumors can certainly be a risk factor for patients with suicide ideation and suicide attempts (Mofatteh, 2021). Damage to the frontal lobe appears to affect divergent thinking, which includes flexibility and problem-solving skills. Additionally, research suggests that attention and memory may continue to be impacted even after significant recovery from a traumatic brain injury or surgery. A notable consequence of frontal lobe damage is a pronounced alteration in social behavior (Jumah and Dossani, 2024; Reber and Tranel, 2019). These are other important matters that need dedicated study in future investigations.

In this study, researchers identified the left superior frontal gyrus (dorsolateral), the right superior frontal gyrus (orbital part), and the right median cingulate and paracingulate gyri as regions with significantly different features extracted from all connectivity measures (ReHo, DC, fALFF, and VMHC). These regions belong to the DMN and the central ECN (frontoparietal network), which have been previously linked to psychiatric disorders characterized by emotion dysregulation such as major depressive disorder (MDD) and suicidal ideation. The study confirms the significant statistical difference between MDDs with and without suicidal ideation and suicide attempters, further emphasizing the importance of these networks in predicting and identifying suicidal ideation.

The study also highlights the reliability of the results as the same regions were found for all measurements and the corresponding features were permuted 10,000 times, demonstrating a consistent and significant difference between the three groups.

It is important to note that this study utilized maps and classification methods to identify regions that can predict suicidal ideation and separate it from non-suicidal ideation, which has not been commonly used in previous studies. Overall, these findings underscore the critical role of the DMN and ECN in understanding and predicting suicidal behavior.

## 5 Limitations

One limitation of our study is the small sample size, which may increase type II errors and limit generalizability. To address this limitation, future studies should utilize larger datasets to enhance statistical power. In the current study, this limitation was partially compensated for by a rigorous feature selection process, independent sample validation of the classification model, and augmenting the training dataset. High accuracy was obtained by considering three groups which makes classification more complicated but more generalized.

Another shortcoming of our study is the inability to explore the long-term evolution of suicidality or predict its course. A follow-up study could examine changes in brain connectivity in individuals with suicidal ideation that may precede a suicide attempt. Additionally, investigating the effects of noninvasive clinical treatments, such as transcranial magnetic stimulation (TMS), may provide valuable information for both female individuals with and without suicidal ideation, including those who have attempted suicide. Furthermore, we only studied female MDD patients with suicidal ideation, considering both males and females would be important for generalizing the results. Subsequent studies adopting similar procedures should include both sexes to improve the comprehensiveness of the findings. Another suggestion is to see whether integrating various subtypes of features enhances the classification process. This should be examined in similar studies with larger sample sizes.

## 6 Conclusion

In conclusion, our study employed machine learning algorithms and feature selection to identify significant brain regions that distinguish MDD patients with and without suicidal ideation (SI). We utilized four different connectivity measurements as features to increase the reliability of our results by identifying commonalities among all methods. Our study identified abnormalities in the Frontal_Sup_Orb, Frontal_Sup, and Cingulum_Mid brain regions as potential neurobiological biomarkers for suicide ideation in individuals with MDD. These areas, along with well-known areas in other standard studies, warrant further investigation to prevent suicide attempts in MDD patients.

Our findings indicate that the DMN and central ECN are the primary brain networks responsible for suicidal thoughts in female MDD patients. These networks contribute to rumination, self-preoccupation, sustained attention, and working memory, highlighting the importance of investigating and examining these functions to prevent suicide attempts in the future.

Future studies should focus on simultaneous clinical measurements (multi-modal data acquisition tools like EEG-fMRI) and task designs, especially in the mentioned areas, to assess risky behaviors such as inhibition and emotion regulation in female MDD patients with or without SI, with a particular emphasis on the DMN and ECN. Dysfunctions in subregions of these networks, such as the superior frontal gyrus and median cingulate gyri, could serve as predictors of risky actions and require further investigation.

## Data Availability

The original contributions presented in the study are included in the article/supplementary material, further inquiries can be directed to the corresponding author.
